# Convergence of *Bar* and *Cry1Ac* Mutant Genes in Soybean Confers Synergistic Resistance to Herbicide and *Lepidopteran* Insects

**DOI:** 10.3389/fpls.2021.698882

**Published:** 2021-10-14

**Authors:** Tien Dung Nguyen, Van Hien La, Van Duy Nguyen, Tri Thuc Bui, Thi Tinh Nguyen, Yeon Ho Je, Young Soo Chung, Xuan Binh Ngo

**Affiliations:** ^1^Department of Biotechnology and Food Technology, Thai Nguyen University of Agriculture and Forestry, Thai Nguyen, Vietnam; ^2^Department of Agricultural Biotechnology, College of Agriculture and Life Science, Seoul National University, Seoul, South Korea; ^3^Department of Genetic Engineering, Dong A University, Busan, South Korea; ^4^Department of Science and Technology for Economic Technical Branches, Ministry of Science and Technology, Ha Noi, Vietnam

**Keywords:** *Cry1Ac* mutation 2, herbicide resistance, *Lepidoptera* insects, soybean transformation, VX93

## Abstract

Soybean is a globally important crop species, which is subject to pressure by insects and weeds causing severe substantially reduce yield and quality. Despite the success of transgenic soybean in terms of *Bacillus thuringiensis* (*Bt*) and herbicide tolerance, unforeseen mitigated performances have still been inspected due to climate changes that favor the emergence of insect resistance. Therefore, there is a need to develop a biotech soybean with elaborated gene stacking to improve insect and herbicide tolerance in the field. In this study, new gene stacking soybean events, such as bialaphos resistance (*bar*) and pesticidal crystal protein (*cry*)*1Ac* mutant 2 (M#2), are being developed in Vietnamese soybean under field condition. Five transgenic plants were extensively studied in the herbicide effects, gene expression patterns, and insect mortality across generations. The increase in the expression of the *bar* gene by 100% in the leaves of putative transgenic plants was a determinant of herbicide tolerance. In an insect bioassay, the *cry1Ac*-M#2 protein tested yielded higher than expected larval mortality (86%), reflecting larval weight gain and weight of leaf consumed were less in the T1 generation. Similarly, in the field tests, the expression of *cry1Ac*-M#2 in the transgenic soybean lines was relatively stable from T0 to T3 generations that corresponded to a large reduction in the rate of leaves and pods damage caused by *Lamprosema indicata* and *Helicoverpa armigera*. The transgenic lines converged two genes, producing a soybean phenotype that was resistant to herbicide and lepidopteran insects. Furthermore, the expression of *cry1Ac*-M#2 was dominant in the T1 generation leading to the exhibit of better phenotypic traits. These results underscored the great potential of combining *bar* and *cry1Ac* mutation genes in transgenic soybean as pursuant of ensuring resistance to herbicide and lepidopteran insects.

## Introduction

Soybean is one of the most important oilseed crops and its growth in many regions of the world is subject to pressure by insects, which is the main factor affecting the yield and quality. Insect pests cause 20–30% biomass losses in soybean (*Glycine max* L.) worldwide (Bengyella et al., 2018). In the Lepidoptera order, *Helicoverpa armigera* Hubner (*H. armigera*) is the main insect pest of several crops such as cotton, chickpea, and soybean (Homrich et al., 2012; Martins-Salles et al., 2017). The application of chemical insecticides is a common measure to control insect pests in soybean fields. However, this practice has raised many problems in terms of the environment and human health. Genetically modified (GM) crops could be an alternative to minimize the application of chemical pesticides. The GM crops express an insecticidal gene to control major lepidopteran insects. This approach not only provides an effective alternative tool for insect management but also addresses other issues such as environmental protection and economic advantages to farmers. In 2017, soybean varieties harboring insecticidal traits stacked with herbicide tolerance were grown in ~95.9 million ha worldwide with an economic value of up to USD 17.6 billion (ISAAA, 2017).

The insecticidal crystal protein (ICPs) imparts resistance against lepidopteran insects. Synergistic activities among different ICPs to augment insect pest resistance have hitherto been reported (Xue et al., 2014). Due to high specificity for exclusive receptors on the membrane of the insect gut epithelial cells (Carroll et al., 1997; Badran et al., 2016), ICPs are shown to be harmless to non-target insects and are compatible with integrated pest management (IPM) systems (Naranjo, 2011). Thus, the expression of ICPs in commercialized *Bacillus thuringiensis* (*Bt*) soybean is a highly important agronomic trait. However, *Bt* soybean varieties have not yet been fully commercialized. Genetic engineering is an important technique to develop *Bt* crops with the pesticidal crystal protein (*cry*) gene (Homrich et al., 2012; Bengyella et al., 2018). The *cry1Ac* gene is one of the *cry* genes isolated from the bacterium *Bt*, and this gene codes for an insecticidal crystal protein. There are several successful reports of genetic transformation into soybean (Yu et al., 2013), cotton (Wei et al., 2003), and sugarcane (Gao et al., 2016). Evidence from laboratory bioassay (Stewart et al., 1996) and field experiments (Walker et al., 2000) shows that transgenic soybean lines with high expression levels of the *cry1Ac* gene significantly increase larval mortality. However, *Bt* transgenic plants often show variable resistance responses against insect pests because of lower *cry* genes expression (Martins-Salles et al., 2017). Thus, there is a need to develop transgenic lines with elaborated gene stacking to limit the emergence of insect resistance. Furthermore, recombinant fusion proteins and domain swapping can be leveraged to protection against insects (Nachimuthu and Kumar, 2004; Martins-Salles et al., 2017). Hence, the challenge is to develop insect-resistant lines by increasing and stabilizing the level of the *cry1Ac* gene in target tissues or use a fusion protein to confer complete protection against insects.

In this study, our goal was to improve the lepidopteran insect resistance in a Vietnamese soybean, by introducing a newly released high expression of *cry1Ac* mutant (M#2). We recommended herein that bialaphos resistance (*bar*) and *cry1Ac*-M#2 genes transferred to soybean offer an enhanced herbicide and insect resistance. The elevated resistance was identified by screening and analyzing plant phenotypes, *cry1Ac* mutant transcriptional level, Southern blotting, and larval insect responses in laboratory bioassays.

## Materials and Methods

### Materials

The plasmids pOB, pENTRTM/D-TOPO, pCambia 3301, and pB2GW7 were sourced from the Invitrogen (Thermo Fisher Scientific, MA USA 02451, www.thermofisher.com). *Escherichia coli* (*E. coli*) strain DH5α and *Agrobacterium tumefacines* (*A. tumefacienes*) strain EHA105 were provided by Prof. Young Soo Chung, Dong-A University, Busan, South Korea. The *cry1Ac* mutants in pOB-Mutant-*cry1Ac*-M#2 (Supplementary Figures 1–3) were developed by a collaboration between the Department of Biotechnology and Food Technology, Thai Nguyen University of Agriculture and Forestry, Vietnam, and Dong-A University and Seoul University, South Korea. The soybean cultivar VX93 used for transformation was provided by the Genetic Institute and Maize Research Institute, Ha Noi, Vietnam.

### Vector Construction

For the construction of binary vector, pOB-Mutant-*cry1Ac* was originally derived from pIM-Mod-*cry1Ac* with Xba I and Bgl II restriction enzymes for *cry1Ac* mutation region (821 bp). The *cry1Ac* mutant gene was constructed by integrating it into the pOB vector. The pOB vector harboring the *cry1Ac* mutant gene, AcNPV 3′-5′ homologous regions, and AcNPV Polh promoter finally constructed pOB-Mut-*cry1Ac* (8,085 bp) (Supplementary Figure 2). Cassettes containing the *bar* and *cry1Ac-*M#2 genes, 35S promoter, and 35S terminator (T35S) were digested from the pOB-Mutant-*cry1Ac* and pENTR^TM^/D-TOPO vector using restriction enzymes, *Bam*HI and *Eco*RI (Invitrogen, USA) (**Figure 7A**, Supplementary Figure 3). The expression vector pB2GW7 was digested by *Bam*HI and *Eco*RI and linked to the cassette with T4-DNA ligase containing the *cry1Ac-*M#2 (Supplementary Figure 4). The mutation of *cry1Ac-*M#2 was confirmed by sequencing and comparing it with the *cry1Ac* gene available in GenBank (https://www.ncbi.nlm.nih.gov/genbank) (Supplementary Figure 5; Tables 1, 2).

### Genetic Transformation and Screening

Genetic transformation of soybean used the “half seed” method described in the study by Olhoft et al. (2003), with minor modifications. Briefly, mature soybean seeds were sterilized with chlorine gas prepared by mixing 3.5 ml of 12 N HCl and 100 ml bleach (5.25% sodium hypochlorite) for 24 h and the sterilized seeds were thoroughly rinsed with sterilized water. From a seed, one cotyledonary node was removed and cut apart of the hypocotyl. The epicotyl was subsequently removed and both axillary bud and cotyledonary node were wounded by scratching with a blade. Then, 50 explants were inoculated in the 50 ml co-cultivation (CC) *A. tumefaciens* suspension (OD = 0.8). Afterward, it was rapidly sonicated for 30 s and vacuumed for 30 s. After 30 min, 10 explants were placed on a solid CC medium of Acetosyringone (0.2 mM) and inoculated at 25°C for 5 days under 16 h light period per day. After this time, excess *A. tumefaciens* were removed from the explants by liquid shoot induction medium (SIM) prepared by adding cefotaxime (250 mg L^−1^) and ticarcillin (250 mg L^−1^). Explants were then transferred into solidified SIM1 containing phosphinothricin (PPT) (10 mg L^−1^) and grown at 25°C under fluorescence lighting 200 μmol photons m^−2^ s^−1^ in 16/8 h light/dark cycles. After 14 days, the newly developed shoots were sub-cultured to fresh SIM2 containing 5 mg L^−1^ PPT for the selection of transformed shoots, and this culture and selection process were continued up to 28 days in shoot elongation medium (SEM) containing PPT 5 mg L^−1^. When the elongated shoot length was over 4 cm, it was transferred to a rooting medium (RM) supplied with indol-3-butyric acid (IBA) at the rate of 0.5 mg L^−1^ for new root induction. After 14 days, T0 plants were grown in the greenhouse until maturity under natural light and 80% relative humidity at 28 ± 2°C.

### Screening of Transgenic Plants Using Herbicide Biochemical Test

Screening the glufosinate-resistant transgenic plants by leaf painting method was carried out as described in the study of by Paz et al. (2004). Briefly, plants with four trifoliate and R1 (early fruiting stage) were screened for putative transformants that expressed the bar gene. The upper surface of a leaf was painted with PPT along with the midrib using a cotton bud (Q-tip). The concentration of active ingredient PPT 0.3, 0.5, 0.7, and 1 mg ml^−1^ was tested. The plant was scored based on the tolerance of leave tissue at 3–5 days after painting.

Under field test, the transgenic and control (non-transgenic) plants were grown in a field experiment at the Thai Nguyen University of Agriculture and Forestry, Thai Nguyen, Vietnam (22°03′ N, 106°14′ E). The randomized completed block design with four blocks of 300 m^2^ (15 × 20 m) each was used to grow plants. Seeds were planted in eight random subsections of each block with two transgenic lines separated by the lines of the control plants. The plants were grown under natural temperature, light, and humidity conditions during the season. Furthermore, 30-day-old plants were preliminarily screened using PPT 0.5 mg ml^−1^ and sprayed with a chemical herbicide (Basta 0.3%, Bayer, Germany) based on a preliminary test referring to the previous study (Paz et al., 2004). After 5 days, the occurrence of yellow leaves and plant death were evaluated. The survival of plants with maintaining green leaves exhibited herbicide tolerance. The following agronomic traits were also measured: plant height, pods per plant, seed number per plant, and thousand-seed weight, and yield.

### Insect Bioassay

An insect bioassay was conducted with *Lamprosema indicata* (F.) larvae based on the previous method of using an artificial diet (Gupta et al., 2004; Singh et al., 2016). Four-day-old *L. indicata* larvae were used for assessing the feeding behavior on leaf tissue of the selected transgenic lines. The young and tender soybean leaflets were clipped from three transgenic lines and non-transgenic plants (control) at 40 days old. One complete leaf was placed on a petri dish on a moistened filter paper. For the insect feeding bioassay, five four-day-old larvae were carefully released on the soybean leaflet in each petri dish to assess the sensitivity of *L. indicata* to the protein encoded by the transferred *cry1Ac* mutant in the leaf tissue. The lids were perforated with a paper pin to ensure air circulation in a room set at 27 ± 1°C, with a relative humidity of 65 ± 5% and a 16-h light/8-h dark cycle. The percentage mortality was calculated from 72 h on daily basis (Abedi et al., 2014). After 3 days, the remaining leaves were evaluated to determine the amount of leaf consumed (g) by the neonates. Similarly, the larval weight gain (g), the percent mortality, and the weight of the leaf consumed on the 3 days were recorded.

### Confirmation of *cry1Ac* Gene Mutant in Transgenic Plants by RT-PCR

Total RNA was isolated from 0.1 g young leaf of PPT- and basta-resistance plants by using customized plant RNAiso Plus Kit (Takara, Takara Bio Inc, Japan, https://www.takara-bio.com/). The cDNA was synthesized using the GoScript Reverse Transcription System (Promega, USA, https://worldwide.promega.com). The presence of the *bar* and *cry1Ac* genes in the putative transgenic soybean were confirmed by RT-PCR using specific primer sequences (Supplementary Table 3). The thermal amplification was carried out using 10X SYBR® Premix Ex TaqTM buffer (including Taq DNA polymerase 5U, Takara, Takara Bio Inc, Japan, https://www.takara-bio.com/), 2.5 mM dNTP mix, 10 pM primers, in 20 μl volume. The RT-PCR reaction condition was as follows: initial denaturation at 95°C for 5 min, followed by 35 cycles of denaturation at 95°C for 30 s, annealing at 55°C for 60 s, and extension at 72°C for 60 s, with a final extension at 72°C for 2 min, and finally samples were maintained at 4°C. Gene expression levels were quantified *via* real-time PCR by using detection on a Bio-Rad system (Bio-Rad, USA, https://www.bio-rad.com). Products were resolved on 1% agarose gel stained with ethidium bromide in 1X TAE buffer and images were captured by Gel Documentation system (BioRad Gel Doc XR, US). The qPCR reaction was performed in triplicate for each of the three independent samples. Quantification of the relative transcript levels used the 2^−ΔΔCT^ method (Livak and Schmittgen, 2001).

### Southern Blot Analysis

Total DNA was extracted from 0.5 g fresh leaves using the CTAB method (Saghai-Maroof et al., 1984) and the DNA was purified using a genomic DNA purification kit (Thermo Fisher Scientific, USA). Southern blot analysis was undertaken following the method of Wang (2005). To produce unique fragments for T-DNA insertion from the pB2GW7 vector, 15 μl total DNA was digested with restriction enzymes *Bam*HI and *Eco*RI. The products were separated on a 1.5% (w/v) agarose gel and blotted onto Amersham Hybond^TM^–N^+^ membrane (GE Healthcare products, Sigma-Aldrich, USA) according to the instructions of the manufacturer. The membrane was prehybridized at 65°C for a minimum of 2 h in 100 ml of 10X SSC solution, pH = 7 (50 M NaCl, 25 mM Na_3_Citrat, 1% SDS) and 100 ml of hybridization solution 20X SSPE, pH = 7.7 [mM NaCl 100, 20 mM NaH_2_PO_4_, 10 mM EDTA, 2% (w/v) SDS]. The probes for *bar-* and *cry1Ac-*M#2 genes-labeled dUTP-11-biotin were inoculated with the hybridization membranes at 65°C. After 4 h, the hybridization membrane was washed with 10X AP solution, pH = 7.5 (40 mM Tris HCl, 25 mM NaCl, 10 mM MgCl_2_) and inoculated with 10 μl of streptavidin alkaline phosphatase (Promega MADISON, WI, USA) in a Blocking solution (Thermo Fisher Scientific, USA) at 37°C for 30 min, then washed again using 10X AP solution. The expected band was detected by inoculation with 200 μl NBT/BCIP solution and the reaction was terminated by 0.5 M EDTA.

### ELISA Analysis of cry1Ac-M#2 Protein in Transgenic Lines

The cry1Ac-M#2 protein content in putative transgenic soybean progenies was measured by using a quantitative ELISA Kit (Envirologix, USA) based on a double-antibody sandwich enzyme-linked immunosorbent assay. Total soluble protein was isolated from young leaf tissue using bicarbonate buffer [15 mM sodium carbonate, 35 mM sodium bicarbonate, 0.1% TritonX 100, 0.05% Tween 20, 1% polyvinylpyrrolidone (PVP), and 1 mM phenylmethanesulfonylfluoride (PMSF)]. The ELISA was performed according to the instructions of the manufacturer (QuliPlate^TM^ Kit for cry1Ab/cry1Ac, Envirologix, Cat. No AP003 CRBS). *Cry1Ac*-M#2 specific primary antibody and second antibody were incubated with soluble protein in each well of ELISA plate. The absorbance of the samples was measured at 405 nm wavelength in ELISA reader (Synergy H1 Hybrid Reader, Biotek, Korea). The quantification of the *cry1Ac*-M#2 protein was based on absorbance values of these protein test samples onto the standard curve of purified cry1Ac-M#2 protein-extracted from *E. coli* DH5α.

### Statistical Analysis

A randomized complete block design was used with five replicates for field trial and sampling date. Moreover, ANOVA was applied to all data. Duncan's multiple range test was employed to compare the means of separate treatments. All statistical tests were performed using SAS 9.1 (SAS Institute Inc., Cary, NC, USA 2002–2003, https://www.sas.com/), and the differences at *P* < 0.05 were considered significant.

## Results

### Production of Transgenic Lines Expressing the *bar* and *cry1Ac*-M#2 Genes

**Figure 1:**
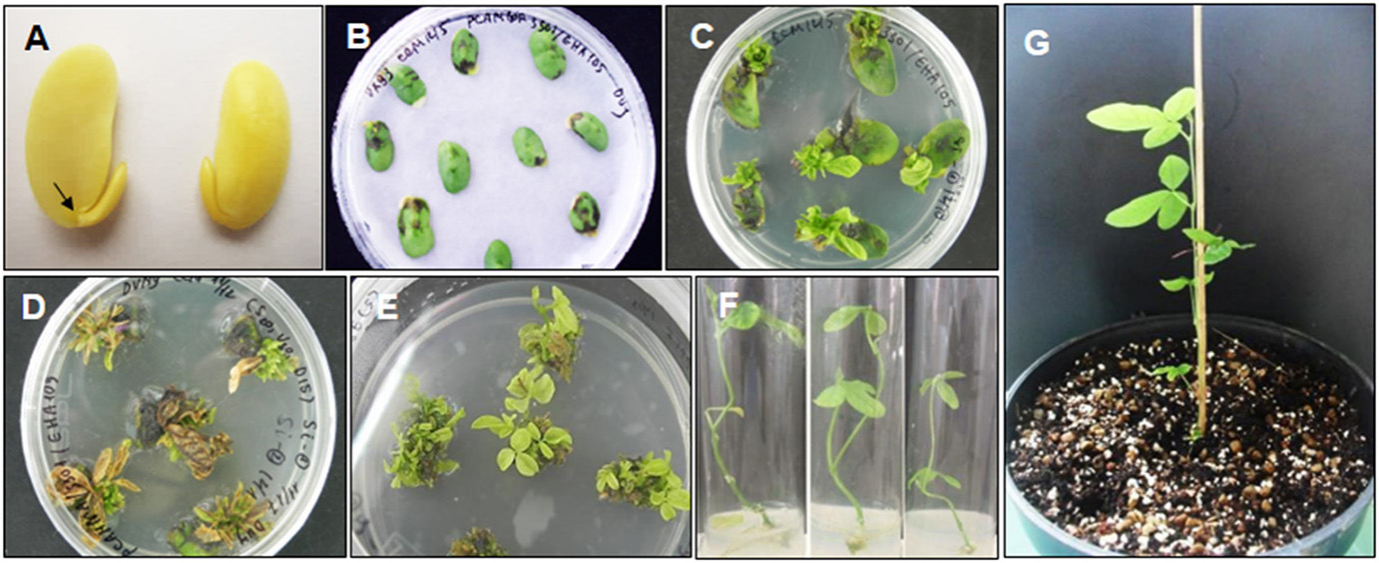
VX93 soybean transformation bearded *bar* and *cry1Ac*-M#2 genes. **(A)** Half seed inoculated Agrobacterium tumefaciens on CCM medium with Whatman filter paper 3MM, **(B)** Shoots induction on SIM media, **(C)** The first selection shoots transgenic in SEM medium with phosphinothricin (PPT) 10 mg L^−1^, **(D)** The second selected shoot-resistance PPT 10 mg L^−1^ in SEM medium, **(E)** Root induction in RM medium, **(F, G)** transgenic plants in soil. Values are represented as mean ± SE (*n* = 3).

**Figure 2:**
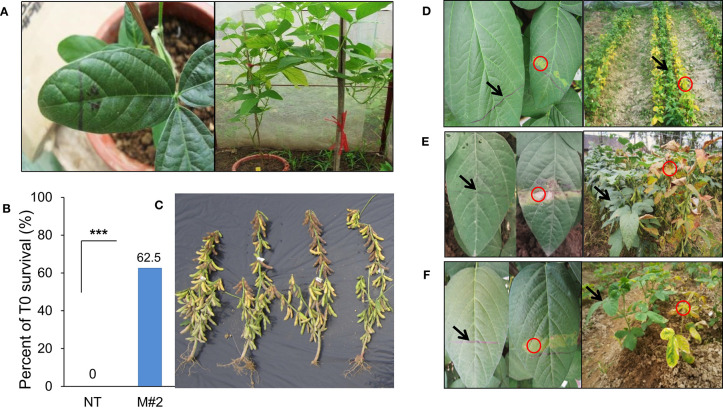
Evaluation of herbicide tolerance from T0 to T3 generation. Three days visualized the morphology of leaf response to PPT.5% following line marker and sprayed basta 0.3% for VX93 transgenic plants in the field after 5 days. **(A)** T0 generation assessed PPT.5 mg mL^−1^. **(B)** T0 survival. **(C)** T0 plants. **(D–F)** T1 to T3 generations tested herbicide tolerance by PPT 0.5 mg mL^−1^ and basta 0.3% in the field, respectively. Arrows indicate the PPT/basta resistant leaves of transgenic plants and circles are the PPT/basta sensitive leaves of control plants. ***P < 0.001.

In this study, we validated the ability to receive *bar* and *cry1Ac*-M#2 genes of a commercial Vietnamese soybean variety VX93. *Cry1Ac*-M#2-mediated *A. tumefaciens* using genetic transformation was attempted to VX93. *Cry1Ac-*M#2 gene-optimized for transformation was constructed with nucleotide sequences that were encoded by T-DNA fragment (**Figure 7A**). In this process, the construct-harbored *bar-* and *cry1Ac*-M#2 were transferred to cotyledonary explants (Figure 1A). A total of 4,740 explants were used for transformation, of which 214 new shoots were developed on the selection medium (Figures 1D,E), with a transformation efficiency of 4.51% (Table 1). Eight putative transgenic plants of T0 generation were grown in the soil (Figures 1F,G) and tested for their PPT resistance ability. Out of eight, five plants showed survival in the different concentrations of PPT range from 0.3 to 1 mg ml^−1^. The leaves of the resistant plants maintained green color after 3 days of PPT painting (Figure 2A). The frequency of green leaf in the putatively transformed lines was 62.5% higher than that in the control lines (Figures 2B,C; Table 2).

**Table 1:** The transformation efficiency of *cry1Ac*-M#2-mediated *Agrobacterium tumefaciens* transferred to VX93 soybean cultivar.

**Soybean cultivar**	**No of explants**	**Percent shoot induced(%)**	**Shoots survival in phosphinothricin selection medium (shoot)**	**Percentage selection (%)**	**Transgenic line grown in greenhouse**
*VX93-cry1A(c)-M#2*	1,871	79.00	46	3.11	3
	1,126	95.20	82	7.65	2
	1,743	87.89	86	5.61	3
	4,740	86.08	214	4.51	8

**Table 2:** Evaluation of the transformation efficiency of VX93-*cry1Ac-M#2* in T0 herbicide-resistance gene (*bar*) by testing phosphinothricin (PPT).

**T0 regeneration**	**No**.	**Transgenic lines phenotype response** **to PPT (mg mL**^**−1**^**)**			
		0.3	0.5	0.7	1.0
*VX93-cry1A(c)-M#2*	1	–	–	–	–
	2	–	–	–	–
	3	+	+	+	+
	4	–	–	–	–
	5	+	+	+	+
	6	+	+	+	+
	7	+	+	+	+
	8	+	+	+	+
VX93 Non-transgenic	NT	–	–	–	–

*Leaf number 4 was used to test different concentrations of PPT. After 3 days, the obtained yellow leaf is negative (–), while others keeping stable blue color leaf is positive (+)*.

### Herbicide Tolerance Revealed the Efficiency Transformation From T1 to T3 Generations

In the field trials, the plants of T1 to T3 generations were tested PPT and Basta, positive and exhibited stable green leaves, whereas all the non-transgenic plants exhibited yellow leaves followed by leaf death after 7 days of treatment (Figures 2D,E). Similarly, T1 generation-putative transgenic plants exhibited the highest survival with 100% plant resistance which gradually decreased in T2 and T3 generations (Table 3).

**Table 3:** Evaluation of the potential ability of VX93 transgenic lines to herbicide resistance (Basta 0.3%) in T1 to T3 generations under field condition.

**Progenies**	**No. plant resistant to basta 0.3%**	**No. plant non-resistant to basta 0.3%**	**Percent of plant resistance (%)**
**T1**			
Transgenic	84^***^	0	100.0^***^
Non-transgenic	0	88	0.0
**T2**			
Transgenic	873^***^	54	94.2^***^
Non-transgenic	0	927	0.0
**T3**			
Transgenic	456^***^	121	79.0^***^
Non-transgenic	0	436	0.0

*Asterisks indicate significant differences between the non-transgenic (control) and transgenic plants;*

*** *P < 0.001*.

**Figure 3:**
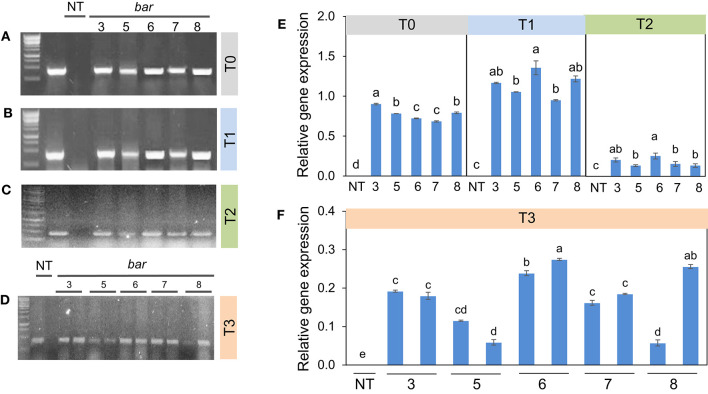
*Bar* gene expression in transgenic plants from T0 to T3 generations. Among these 3, 5, 6, 7, 8 are transgenic lines and non-transgenic plants (NT). *Bar* gene expression of five plants **(A–C)**, left to the right border, in which lane 1 (marker 1kb), lane 2 (plasmid contained pB2WG7 vector), lane 3 negative plant (VX93 non-transgenic, NT), lanes 4 to 8 T0 and T2 generation, respectively. **(D)** left to right border, it showed 10 plants in the T3 generation (from lanes 4 to 13), therein lane 1 is maker 1 kb, lane 2 is plasmid contained pB2WG7 vector, lane 3 is a negative plant (VX93 non-transgenic), and lanes 4 to 13 are transgenic plants. Relative gene expression of T0 to T2 **(E)**, and T3 **(F)** for each plant consistently.

**Figure 4:**
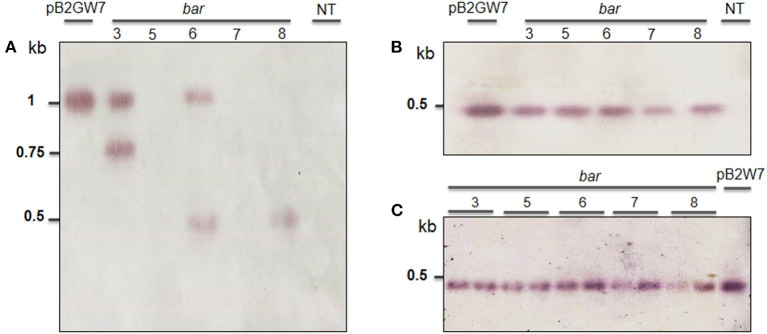
Southern blot analysis of T1 to T3 transgenic plants obtained from *Agrobacterium*-mediated transformation of VX93 cultivar. The DNA soybean transgenic plant was cut by *Sac*I enzyme. The process used the probe-*bar* gene expression in NBT-BCIP. The figures **(A, B)** indicated T1 and T2 generation (left to right), lane 1 is pB2GW7 vector, lane 2 to 6 (five plants), lane 7 is a non-transgenic plant (NT). **(C)** T3 generation (left to right), lanes 1–10 (ten plants), and lane 11 is pB2GW7 vector. Plants were chosen randomly for southern blot analysis.

These results are consistent with the RT-PCR and validated that the transgenic plants induced herbicide tolerance. *Bar* gene was remarkably expressed in T0 (Figure 3A), accompanied by relative gene expression. A much higher *bar* gene expression was randomly observed in the T1 generation (Figure 3E). However, the intensity of this gene expression was largely decreased in T2 and T3 generations (Figures 3E,F). On the other hand, locus integration in genomic DNA digested with *Sac*I enzyme was detected in southern blot analysis. As expected, the *cry1Ac*-M#2 transgenic lines resistance to PPT was positive for hybridization with the probe *bar* and *cry1Ac* mutant 2 genes (Figure 4A). Notably, a single band (0.5 kb) was detected in all of the transgenic events corresponding to homozygous in T2 and T3 generations, respectively (Figures 4B,C).

### Expression of *cry1Ac*-M#2 Reduced the Negative Effects of Insect

**Figure 5:**
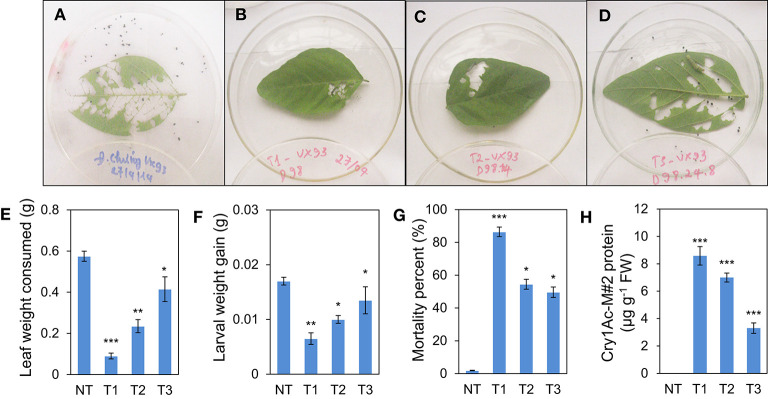
Insect feeding bioassay of transgenic lines No. 98. **(A)** Detached leaf assay in VX93 non-transgenic **(A)** and transgenic line (VX93-M#2) from T1 to T3 generations **(B–D)**, respectively. **(E)** average leaf consumed by neonates in all transgenic lines T0 to T3 compared with non-transgenic VX93 (control). **(F)** Lower average larval weight gain. **(G)** Significant higher percent mortality of neonates in transgenic VX93 line (T1 to T3 generations) compared to VX93 non-transgenic (NT). **(H)** Cry1Ac-M#2 protein content detected by Elisa analyzing. Values are represented as mean ± SE (*n* = 3). Asterisks indicate significant differences between the control and pathogen-stressed plants; *P < *0.0*5, **P < *0.0*1, ***P < *0.0*01.

After 72 h, the detached leaf bioassay showed that the weight of leaf consumed was lower in T1-transgenic lines than in T2 and T3 generations (Figures 5A–E). Similarly, larval weight gain was the lowest in the *Bt*-soybean transgenic line of T1 generation, i.e., 38.2% fewer larvae weight gain compared with the non-transgenic plants (*P* < 0.001). Consequently, the leaf weight consumed of T1 plants was less than that of T2 and T3 generations (Figure 5E). In contrast, the survival of leaf roller (*L. indicata*) larvae was significantly different when fed the leaves from transgenic lines of T1 to T3 generations. Indeed, the percentage of larval mortality was significantly increased in transgenic plants of T1 generation with a rate of 86.5% compared with non-transgenic plants (*P* < 0.001), whereas it was slightly decreased in T2 and T3 generations (*P* < 0.05) (Figure 5G). Similar to the pattern of the cry1Ac-M#2 protein content was detected in T1 to T3 generations (Figure 5H).

### *Cry1Ac*-M#2 Induced Insect Resistance Under Field Conditions

Under field conditions, the rate of insect resistance observed in T1 to T3 generations was significantly different between transgenic and non-transgenic lines. Generally, *L. indicata* and *H. armigera* were affected to a greater extent in the T2 and T3 generations compared with the T1 generation (Table 4). Transgenic plants were more resistant to *L. indicata*, considering the significantly lower rate of leaf damage (*P* < 0.05) and less than four-fold of the rate of plant damage in T2 (*P* < 0.001) compared with non-transgenic lines. However, no significant difference between transgenic and non-transgenic lines was observed on the rate of plants damaged by *H. armigera* insect in the T1 generation, but it was less in T2 and T3 generations.

**Table 4:** Main insect effect of VX93 transgenic lines from T1 to T3 generations under field condition.

**Progenies**	***Lamprosema indicata***		***Helicoverpa armigera***	
	**Rate of plant damage (%)**	**Rate of leaves damage (%)**	**Rate of plant damage (%)**	**Rate of pod damage (%)**
**T1**				
Transgenic	0.00^*^	4.12^*^	1.19	3.25^*^
Non-transgenic	2.10	5.38	2.38	5.80
**T2**				
Transgenic	2.00^***^	5.30^*^	2.40^**^	2.10
Non-transgenic	8.00	7.05	8.33	3.03
**T3**				
Transgenic	9.20^*^	4.40^*^	6.20^*^	3.00^*^
Non-transgenic	13.33	6.85	8.20	5.61

*Asterisks indicate significant differences between the non-transgenic (control) and transgenic plants;*

* *P < 0.05*,

** *P < 0.01*,

*** *P < 0.001*.

**Figure 6:**
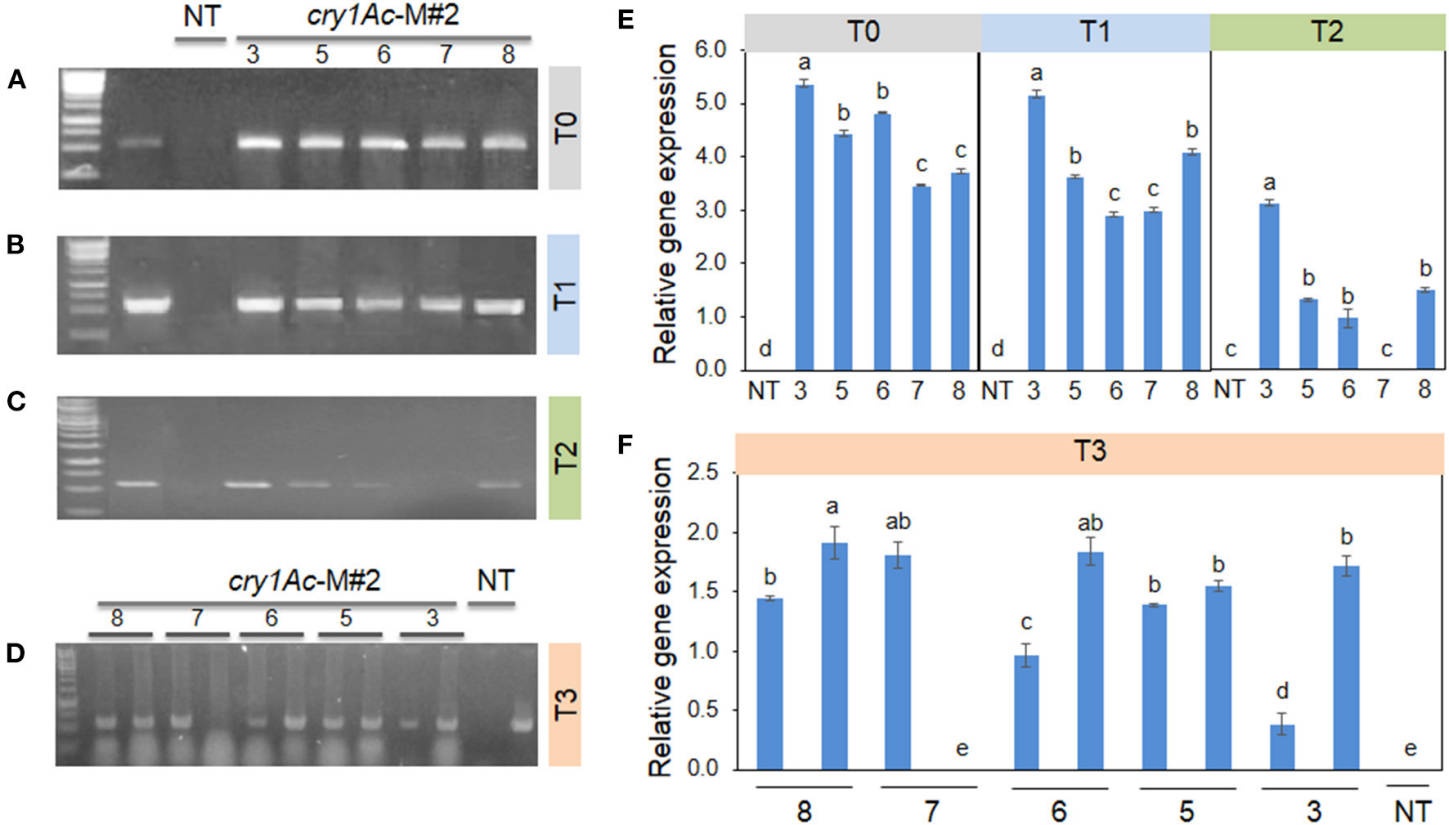
*Cry1Ac* mutant (M#2) expression in transgenic plants from T0 to T3 generations. Among these 3, 5, 6, 7, 8 are transgenic lines and non-transgenic plants (NT). *Cry1Ac* mutant (M#2) gene expression of five plants **(A–C)** left to the right border, in which lane 1 (marker 1kb), lane 2 (plasmid contained pB2WG7 vector), lane 3 negative plant (VX93 non-transgenic, NT), lanes 4 to 8 T0 and T2 generation for cry1Ac mutant (M#2), respectively. **(D)** showed 10 plants in the T3 generation (from lanes 2 to 11), therein lane 1 is maker 1 kb, lane 12 is a negative plant (VX93 non-transgenic), and lane 13 is plasmid contained pB2WG7 vector). Relative gene expression of T0 to T2 **(E)**, and T3 **(F)**.

**Figure 7:**
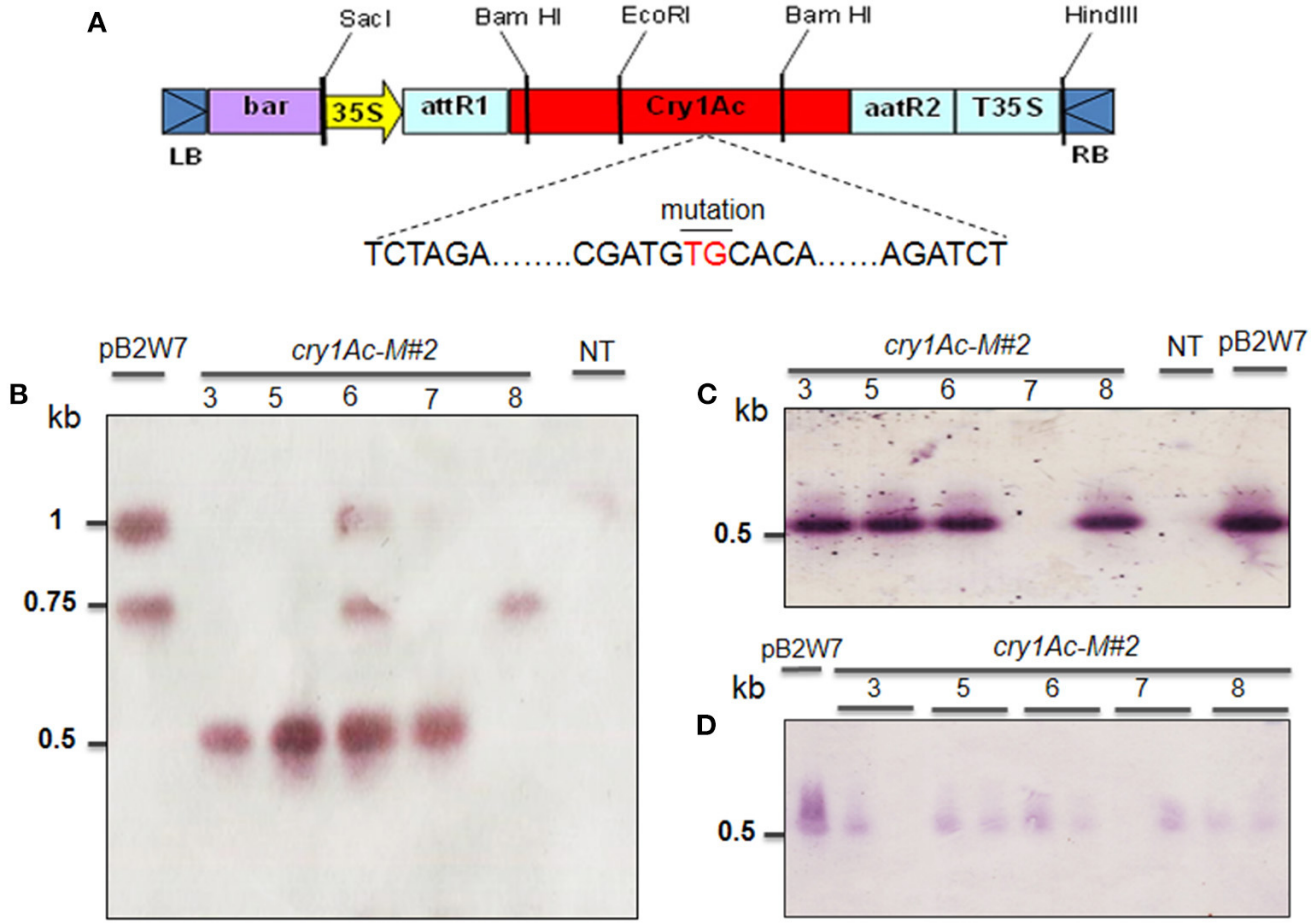
Southern blot analysis of transgenic plant in T1 to T3 generations obtained from *Agrobacterium*-mediated transformation of VX93 cultivar. The DNA soybean transgenic plant cut by cut by *Bam*HI and *Eco*RI enzymes. The process used the probe-bar gene expression in NBT-BCIP. **(A)** T-DNA structure harbored *bar* and *cry1Ac-M#2* genes (TG nucleotides mutation, red color indicator). **(B)** T1 generation (left to right), lane 1 is pB2GW7 vector, lane 2 to 6 (five plants), lane 7 is a non-transgenic plant (NT). **(C)** T2 generation (left to right), lane 1 to 5 (five plants), lane 6 is a non-transgenic plant (NT), and lane 7 is the pB2GW7 vector. **(D)** T3 generation (left to right), lanes 2–11 (10 plants), and lane 1 is pB2GW7 vector. Plants were chosen randomly for southern blot analysis.

These results were confirmed by the presence of *cry1Ac* mutant expression in *Bt*-soybean lines. The *cry1Ac* expression levels of *Bt*-soybean were significantly different compared with the non-transgenic plants. The expression pattern of *cry1Ac*-M#2 in five transgenic plants of T1 and T2 generations was similar (Figures 6A,B). However, the result in Southern blot digested by *Bam*HI and *Eco*RI enzymes (Figure 7A) revealed that four transgenic lines had an expected band of 0.5 kb (Figures 7B,C). Subsequently, the *cry1Ac* mutant expression levels were less in the transgenic line of T2 and T3 generations, whereas *cry1Ac*-M#2-4 was not detected by RT-PCR (Figures 6C–F) and Southern blot (Figures 7C,D) in the T2 transgenic line, similar to one plant in the T3 generation.

### Agronomic Performance of Soybean Transgenic Lines Under Field Conditions

Agronomic traits such as plant height, primary branches, pods per plant, seed per plant, and seed weight of the T1 to T3 transgenic plant were compared with the non-transgenic plants grown in the field (Table 5). Generally, the average height of transgenic plants was greater than that of non-transgenic plants. Indeed, the majority of transgenic plants in the T1 generation exhibited superior plant growth compared with the non-transgenic line, T2, and T3 generations during the experiment period (*P* < 0.05). There was no difference in the primary branches measured in T1 to T3 generations. The yield of mature fruit and numbers of seeds per plant were greater in the T1 and T2 generations. Similarly, seed weight per plant of transgenic plants in the T1 generation was significantly higher than that of the non-transgenic plants (*P* < 0.05).

**Table 5:** Growth and development of VX93 transgenic lines from T1 to T3 generations under field condition.

**Progenies**	**Plant height (cm)**	**Sub-branches**	**Mature fruit per plant**	**Seeds per plant**	**Seeds weight per plant (g)**
**T1**					
Transgenic	100.8^*^	4.0	59.7	118.6	19.3^*^
Non-transgenic	81.0	4.0	57.4	116.0	16.1
**T2**					
Transgenic	80.1	3.5	59.9	131.6	23.1
Non-transgenic	78.0	3.8	57.8	126.0	22.3
**T3**					
Transgenic	87.8	4.2	58.0	119.4	21.4
Non-transgenic	86.0	3.3	60.7	121.0	20.5

*Asterisks indicate significant differences between the non-transgenic (control) and transgenic plants;*

* *P < 0.05*.

## Discussion

Transgenic soybean containing multiple *Bt* genes are conferred with resistance to important insect pests (Romeis et al., 2019). A transgenic lineage of soybean expressing the *cry1Ac* gene has enhanced resistance to *Lepidoptera* insects (Walker et al., 2000; Yu et al., 2013), and further research developed with respect to maintaining insect resistance (Badran et al., 2016; Singh et al., 2016; Romeis et al., 2019). A study showed that *Bt*-soybean with *cry1Ac* expression provided good protection against *H. armigera*, however, limited resistance efficiency was found in transgenic soybean (Yu et al., 2013). Therefore, determining the thresholds of regulatory expression at which the *cry1Ac* gene switches from hypersensitive responses to insect resistance and survival in transgenic soybean would provide valuable insights into insect resistance. Accordingly, one of the aims of the present study was to test the effect of the *cry1Ac* mutants that would accelerate the resistance of the soybean against *Lepidoptera* insects, because this gene produces *cry* protein toxicity for this insect (Romeis et al., 2006). Therefore, this present study assessed preferentially relative transcriptional expression, insect mortality levels, and inheritance of *cry1Ac* mutant.

It has been well documented that the combination of different traits or genes in genetically modified plants has been a trend. It is advantageous to provide desirable characteristics in genetically modified plants, e.g., stacking multiple herbicides and insect resistance in soybean. Among these, the *bar* gene is a highly efficient selectable marker in plant genetic transformation and attribute to plant resistance to herbicides (Gordon-Kamm et al., 1990; Abdeen and Miki, 2009; Yun et al., 2009), which is detoxifying by the phosphinothricin N-acetyltransferase enzyme (Lutz et al., 2001; Yun et al., 2009; Huang et al., 2014). It has been widely used in many plant species, including soybean, due to its advantage in screening putative transformants (Kita et al., 2009). Indeed, in this study, the *cry1Ac* mutant 2 transformation efficiency was released on *bar* gene expression, because the *bar* gene in the T-DNA segment was harbored with *cry1Ac*-M#2 (Figure 7A), accompanied to shoot induction survival of 4.51% (Table 1; Figure 1). Moreover, in the field test, VX93 transgenic exhibited much greater Basta resistance compared with the non-transgenic line (Table 3). Putative transformants surviving in tissue culture or field trial tests could be screened by treatment with PPT or Basta herbicide. This procedure allowed rapid identification of positive transgenic plants because the leaves in tissue cultures of transgenic plants are green (Figure 1E), while non-transgenic leaves turned yellow (Figure 1D), these symptoms were recorded similarly the following spraying with Basta 0.3% (Figure 3). The non-transgenic-induced hypersensitive response was accompanied by yellow leaves and the death of leaves (Figures 3D–F). Severe yellow leaves in the non-transgenic plants reflected no *bar* gene expression. According to VX93, putative transgenic plants from T0 to T3 generations induced PPT resistance-mediated *bar* gene activity (Figures 2, 3), which in turn increased PPT-resistance levels (Table 2). Expression of the *bar* phenotype was not consistent from one generation to the next in only five lines. In the T1 plants 5 and 7, no *bar* expression was detected in leaf tissue by Southern blot (Figure 4A). However, *bar* expression was segregated in the leaf of T2 and T3 plants (Figures 4B,C). Such change in *bar* expression between generations has been reported in soybean Bert (Olhoft et al., 2003) and Jack (Reddy et al., 2003). It is possible that unstable *bar* expression was a result of silencing or elimination between the generations or difference in *bar* expression in the particular tissue analyzed. According to this, several studies have reported that soybean transformation efficiency tends to be low, because of transgene silencing or transgene loss, in which silencing of transgene expression in the progeny plants was reported in 10% of transgenic lines (Vain et al., 2002; Olhoft et al., 2003). Testing the *bar* gene silencing was due to transcriptional or post-transcriptional level. RT-PCR showed that the *bar* gene was expressed in five transgenic lines during T1 to T3 progenies (Figures 3B,E). This result suggested that *bar* transgene silencing in these five transgenic lines may not be due to post-transcriptional gene silencing, similar to what was previously reported (Reddy et al., 2003; Zhang et al., 2006; De Bolle et al., 2007), and *cry1Ac* transgene silencing (Gao et al., 2016). Similarly, the study of Zhenyu et al. (2019) reviewed that the overexpression of the *Bt* gene at earlier stages of transgenic cotton plants resulted in gene regulation at the post-transcription level and caused the gene silencing consequently. Moreover, the increased *bar* gene coincided with the green leaf mediated stable *bar* gene inheritance during T0 to T3 progenies (Figure 3), which significantly reduced leaf toxicity and leaf death (Figure 2), thereby alleviating the negative symptoms-induced by PPT or Basta treatments. Therefore, a significant expression of the *bar* gene was observed in leaves of VX93 transgenic plants, possibly activating regulatory mechanism resistance to PPT or Basta herbicide. Thus, stable inheritance of the *bar* transgene is important to obtain commercially useful soybean transgenic lines.

Several reviews have documented herbicide and insect resistance in transgenic crops that are important agronomic traits (Lutz et al., 2001; Singh et al., 2016; Martins-Salles et al., 2017; Romeis et al., 2019). Useful genes can be introduced to crops without leading to interference with normal plant metabolism (Block et al., 1995; Gao et al., 2016; Gupta et al., 2020). Thus, the simultaneous expression of the *bar* and *cry1Ac* genes has been postulated to be a key to the resistance capabilities of soybean (Kita et al., 2009). Numerous studies have indicated that *cry* genes encoded for *Bt* protein can reduce the effects of insects (Bravo et al., 2011; Lu et al., 2012). A previous study described *cry* gene expression in soybean and observed insecticidal activity, e.g., soybean Jack-*Bt* expressed *cry1Ac* gene exhibited five times less defoliation (Walker et al., 2000), and provided good protection against corn earworm (Yu et al., 2013). Most studies of *Bt* transgenic soybean with a *cry* protein coded by *cry1Ac* gene (Yu et al., 2013), thereby providing a possible *Bt* soybean-mediated *cry1Ac* expression option to regulate *Lepidoptera* resistance. It is well known that *cry* genes produce endotoxins specific to some major insects of important crops (Gatehouse, 2008; Yu et al., 2013). Many *cry* genes have been characterized and tested against insects (Bengyella et al., 2018). However, from the first testing of *Bt* crops to the present, the development of resistance to *cry* toxins in insects has remained a major concern (Tabashnik et al., 2008; Romeis et al., 2019). The much subscribed strategy for delaying resistance development is “high dosage/refuge” (Bates et al., 2005; Gryspeirt and Gregoire, 2012). The success of this strategy depends on using a refuge zone containing non-*Bt* plants susceptible to the insect and *Bt* plants expressing a high concentration of cry toxins. Among these, high dosage *cry* toxins released on insecticidal and closely related to *cry1Ac* gene expression levels (Gao et al., 2016, Singh et al., 2016). Thus, it is important to determine the copy number of transgenes in transgenic plants, because the copy number can affect genetic stability and expression level. Thus, we developed transgenic events with *cry1Ac-*M#2, both containing a nucleotide-modified truncated *cry1Ac* gene (Supplementary Figures 1–3). Furthermore, the expression of *cry1Ac*-M#2 in VX93 transgenic soybean was remarkably increased in the T0 and T1 generations but slightly decreased in the T2 and T3 generations (Figure 6). Meanwhile, quantification of the *bar* and *cry* gene expression level between Southern blotting and RT-PCR in T2 and T3 suggested a generation-dependent pattern (Figures 3, 6), as shown in the significant herbicide- and insect resistance (Tables 3, 4). Among the many networks involved in insect resistance-dependent *Bt* toxin gene expression in a temporal and spatial variation, overexpression of the *cry1Ac* gene at the post-transcription level leading to consequent gene silencing has also been found (Adamczyk Jr et al., 2009). According to the study of Walker et al. (2000), a transgenic Jack-Bt showed greater resistance than untransformed Jack to the natural infestation of lesser cornstalk borer. It should be noted that *cry1Ac*-M#2 expression levels and the copy number of *cry1Ac* gene in T2 and T3 generations (Figures 6C,D and 7C, D) were not detected in RT-PCR and Southern blot, which could result in co-suppression due to multi-copy integration; thus, leading to transgene silencing. Transgenic silencing has hitherto been reported in soybean (Olhoft et al., 2003) and sugarcane (Zhou et al., 2018). The results of several other studies have reported the *Bt*-soybean against insects in regulating *cry1Ac* response, e.g., *cry1Ac*-activated *cry* protein toxin (Walker et al., 2000; Jamil et al., 2021), involvement of insecticidal activity-induced insect resistance (Gao et al., 2016), and *cry1Ac* gene-induced *Lepidoptera* resistance (Yu et al., 2013; Jamil et al., 2021). The insect bioassay revealed a significant increase in the larval mortality rate and larval weight gain of *L. indicata* (Figures 5F,G), but reduced leaf weight consumed (Figure 5E) when compared with that of the non-transgenic plants, as well as coincided with the expression of *cry1Ac*-M#2 gene (Figure 6). Indeed, the highest expression of *cry1Ac*-M#2 in leaves of transgenic soybean occurred in T0 and T1 generations, thereby alleviating the rate of damage and negative symptoms induced by insects (Table 4; Figures 6A–D). It is worth noting that there was a remarkable difference in the insect symptoms of *cy1Ac*-M#2, e.g., leaf weight consumed and larval weight gain, even though plants in both *cry1Ac* mutant expression exhibited a significant inheritance in T0 to T3 generations. These results demand further discussion of *cry1Ac* mutant regulatory insecticidal activity involved in the integrative process of insect resistance, and this should emphasize the most distinct differences in the mortality of insects and *cry1Ac*-M#2 expression in leaves (Figures 6A–F). The variable insect mortality level was mostly in agreement with the *cry* protein-dependent intensity of *cry1Ac* mutant expression levels. In addition, as far as we know, this study provided the first report on the high expression of *cry1Ac*-M#2 increased *Lepidoptera* resistance level. Given that the *cry1Ac* mutant triggers insecticidal activity, specifically induced transgenic soybean defense signaling, it is reasonable to conclude that the *Bt*-VX93 soybean has mediated the overproduction of *cry1Ac* mutant, thereby functioning as crucial regulatory insect resistance.

In the *Bt*-soybean, high-level expression of the *cry1Ac* gene in soybean leaves is important to obtain insect resistance (Yu et al., 2013). In the present study, *cry1Ac-*M#2 expression was highest in VX93 transgenic leaves, but not expressed in the leaves of non-transgenic plants (Figures 5A,B). A significant *Bt* soybean against *Lepidoptera* for *cry1Ac* mutant gene-transduction insecticidal activity was evaluated in insect bioassay (Figures 5, 7). Increasing evidence demonstrates that *cry1Ac* expression is the first plant-produced insecticidal protein and that *cry1Ac* is the master activator of *Lepidoptera* resistance (Walker et al., 2000; Yu et al., 2013; Martins-Salles et al., 2017). In the field evaluation, there was substantively less *Lepidoptera* insect damage on plants, leaves, and pods in the transgenic VX93 compared with non-transgenic plants (Table 4). The insect resistance level and response to insect feeding were observed in the insect bioassay (Figures 5A–D), which was consistent with the pattern of the *cry1Ac* mutant gene (Figures 5C,D and 6). The *cry1Ac* specifically responds to *Lepidoptera* insect, e.g., *L. indicata* and *H. armigera* (McPherson and MacRae, 2009; Tabashnik et al., 2009; Yu et al., 2013; Martins-Salles et al., 2017), thereby providing a venue for soybean transgenic induced insect resistance (Figure 5; Table 4) in high *cry1Ac-*M#2 expression during T1 to T3 generations. These results were higher than that of previous studies in sugarcane (Weng et al., 2006) and soybean (Yu et al., 2013) which were used with normal *cry1Ac*. According to this study, larval mortality was dependent on the highest *cry1Ac-*M#2 expression levels in *Bt*-soybean leaves (Figure 5). In the feeding leaf test, the results indicated that larval mortality exceeded 86% in the T1 generation (Figure 5G), which is higher than *cry1Ac* un-mutation in *Bt*-soybean MON87701 (Yu et al., 2013). Indeed, the study of Yu et al. (2013) reported larval mortality around 76% when fed with transgenic soybean leaves. These results demonstrated that *cry1Ac*-M#2 has been proven more effective than *cry1Ac* non-mutation and this gene is essential for insect control. However, the expression of *cry1Ac*-M#2 in transgenic soybean was declined consistently during the growing period, which confirmed *cry1Ac*-M#2 protein level found in the leaf of transgenic lines from T1 to T3 generations at vegetative stages (before anthesis) (Figures 5G, 6). In accordance with this, leaf weight consumed and larval weight gain significantly increased in T2 and T3 generations (Figures 5E,F), accompanied to the efficiency against *L*. *indicata* in artificially infested active larval mortality (Figure 5G) and *cry1Ac*-M#2 protein lower (Figure 5H). It supported that a high degree of resistance against *Lepidoptera* insects dependent *cry1Ac*-M#2 levels. On the other hand, the study of Weng et al. (2006) reported a modified *cry1Ac* gene in sugarcane ROC16 and YT79-177 had comprised 62% of the transgenic plant which were resistant to stem borer damage in both greenhouse and field trials. The resistance of *Bt* crops to target insects is generally correlated with the levels of insecticidal protein (Walker et al., 2000; Gao et al., 2016; Singh et al., 2016). In the present study, the highest expression of *cry1Ac*-M#2 in transgenic leaves was detected in the vegetative stage (before anthesis), as well as the reduced rate of *L. indicata* and *H. armingera* insects damage (Table 4). Thus, transgenic soybean lines revealed efficacy against these insects feeding in the field. Significant reductions in the larval populations of *L. indicata* and *H. armingera* were observed in transgenic soybean lines expressing *cry1Ac*-M#2 compared with the non-transgenic plants at the field. Similarly, the study of Yu et al. (2013) reported that *Bt*-soybean expressing *cry1Ac* targeted *H. armingera* before anthesis. Compared with the non-transgenic, transgenic soybeans were more efficient to larval mortality in the T1 generation, despite this symptom decreased in T2 and T3 generations. This is in agreement with earlier reports of *Bt*-soybean expressing *cry1Ac* caused high first-instar mortality in *H. armingera* (Yu et al., 2013).

Given that agronomic traits depend on the *cry1Ac* genes, responsive-related genes expression has been reported. The previous study evaluated that the majority yield was not affected by *cry1Ac* expression in transgenic soybean (Homrich et al., 2008) or found that there were no unintended changes in the seed composition of transgenic chickpea-expressed a truncated-*cry1Ac* gene (Gupta et al., 2020). In the present study, agronomic traits in the transgenic plants were similar to the non-transgenic plants (Table 5). However, in the T1 generation, the transgenic plants exhibited variable plant height and seed weight per plant (*P* < 0.05) compared with the non-transgenic plants, but these traits were not different in the T2 and T3 generations. The plant height was higher in *Bt* transgenic than in the non-transgenic plants, which was consistent with the *cry1Ac*-M#2 expression with the greatest effect in the T1 generation (Table 5), thereby providing a possible *cry1Ac* mutant-mediated option to regulated plant growth and development reactions, e.g., leafing speed, branches forming, and burning effective (Chen et al., 2019a). Increasing evidence demonstrates that *cry8-*like gene expressing in *Jinong28* soybean enhances plant growth and yield (Qin et al., 2019). Moreover, there is a synergistic and significant interaction between insect resistance and plant growth for the improvement of seed weight (Tables 3–5), which may potentially interact with carbon and nitrogen metabolism (Coviella et al., 2002; Rochester, 2006) and integrative cellular hormone (GA_3_) regulation processes that promote *Bt* cotton yield (Chen et al., 2019b). However, the mechanisms by which *cry1Ac* mutant-elicited nitrogen metabolism improves plant growth remains unclear and requires further investigation.

In summary, the results of both the present study and previous reports (Zhang et al., 2006; Yu et al., 2013) provided evidence on *Bt*-soybean-mediated *bar-* and *cry1Ac*-M#2 transcriptional response, which may promote herbicidal and insecticidal activity. *Bt*-soybean-mediated modulations were characterized by (1) herbicide tolerance stably inherited in T1 to T3 progeny, (2) negative insect-induced symptoms were largely alleviated in *Bt* soybean, and (3) synergistic interactions occurred between insect resistance, plant growth, and seed yield in *Bt*-soybean.

## Data Availability Statement

The original contributions presented in the study are included in the article/Supplementary Material, further inquiries can be directed to the corresponding author.

## Author Contributions

TDN, VHL, YSC, and XBN designed the experiment, interpreted the data, and wrote the manuscript. TDN, VHL, VDN, and TTB carried out the chemical analysis. TTN carried out the field experiments. YHJ made vector construction. All authors reviewed and approved the submitted version of the manuscript.

## Conflict of Interest

The authors declare that the research was conducted in the absence of any commercial or financial relationships that could be construed as a potential conflict of interest.

## Publisher's Note

All claims expressed in this article are solely those of the authors and do not necessarily represent those of their affiliated organizations, or those of the publisher, the editors and the reviewers. Any product that may be evaluated in this article, or claim that may be made by its manufacturer, is not guaranteed or endorsed by the publisher.
