# Supplementary material for: Convergence of Bar and Cry1Ac Mutant Genes in Soybean Confers Synergistic Resistance to Herbicide and Lepidopteran Insects
Source: Front Plant Sci. 2021 Oct 14;12:698882. doi: 10.3389/fpls.2021.698882 (PMC8559871; doi:10.3389/fpls.2021.698882)
Supplement: Supplementary file 1 [file Data_Sheet_1.pdf]

Supplementary Material

Table S1. Comparison of the sequence *cry1Ac* non-mutation and mutation with the Blast/NCBI

1. Comparison of forward primers sequence and *cry1Ac* gene on GenBank

```
Score = 1818 bits (984)     Expect = 0.0    Identities = 992/995 (99%)
Gaps = 3/995 (0%)           Strand=Plus/Plus

Query  130   ATGGACAACAACCCAAACATCAACGAATGCATTCCATACAACTGCTTGAGTAACCCAGAA  189
             ||||||||||||||||||||||||||||||||||||||||||||||||||||||||||||
Sbjct  1     ATGGACAACAACCCAAACATCAACGAATGCATTCCATACAACTGCTTGAGTAACCCAGAA  60

Query  190   GTTGAAGTACTTGGTGGAGAACGCATTGAAACCGGTTACACTCCCATCGACATCTCCTTG  249
             ||||||||||||||||||||||||||||||||||||||||||||||||||||||||||||
Sbjct  61    GTTGAAGTACTTGGTGGAGAACGCATTGAAACCGGTTACACTCCCATCGACATCTCCTTG  120

Query  250   TCCTTGACACAGTTTCTGCTCAGCGAGTTCGTGCCAGGAGCTGGGTTCGTTCTCGGACTA  309
             ||||||||||||||||||||||||||||||||||||||||||||||||||||||||||||
Sbjct  121   TCCTTGACACAGTTTCTGCTCAGCGAGTTCGTGCCAGGAGCTGGGTTCGTTCTCGGACTA  180

Query  310   GTTGACATCATCTGGGGTATCTTTGGTCCATCTCAATGGGATGCATTCCTGGTGCAAATT  369
             ||||||||||||||||||||||||||||||||||||||||||||||||||||||||||||
Sbjct  181   GTTGACATCATCTGGGGTATCTTTGGTCCATCTCAATGGGATGCATTCCTGGTGCAAATT  240

Query  370   GAGCAGTTGATCAACCAGAGGATCGAAGAGTTCGCCAGGAACCAGGCCATCTCTCGTTTG  429
             ||||||||||||||||||||||||||||||||||||||||||||||||||||||||||||
Sbjct  241   GAGCAGTTGATCAACCAGAGGATCGAAGAGTTCGCCAGGAACCAGGCCATCTCTCGTTTG  300

Query  430   GAAGGATTGAGCAATCTCTACCAAATCTATGCAGAGAGCTTCAGAGAGTGGGAAGCCGAT  489
             ||||||||||||||||||||||||||||||||||||||||||||||||||||||||||||
Sbjct  301   GAAGGATTGAGCAATCTCTACCAAATCTATGCAGAGAGCTTCAGAGAGTGGGAAGCCGAT  360

Query  490   CCTACTAACCCAGCTCTCCGCGAGGAAATGCGTATTCAATTCAACGACATGAACAGCGCC  549
             ||||||||||||||||||||||||||||||||||||||||||||||||||||||||||||
Sbjct  361   CCTACTAACCCAGCTCTCCGCGAGGAAATGCGTATTCAATTCAACGACATGAACAGCGCC  420

Query  550   TTGACCACAGCTATCCCATTGTTCGCAGTCCAGAACTACCAAGTTCCTCTCTTGTCCGTG  609
             ||||||||||||||||||||||||||||||||||||||||||||||||||||||||||||
Sbjct  421   TTGACCACAGCTATCCCATTGTTCGCAGTCCAGAACTACCAAGTTCCTCTCTTGTCCGTG  480

Query  610   TACGTTCAAGCAGCTAATCTTCACCTCAGCGTGCTTCGAGACGTTAGCGTGTTTGGGCAA  669
             ||||||||||||||||||||||||||||||||||||||||||||||||||||||||||||
Sbjct  481   TACGTTCAAGCAGCTAATCTTCACCTCAGCGTGCTTCGAGACGTTAGCGTGTTTGGGCAA  540

Query  670   AGATGGGGATTCGATGCTGCAACCATCAATAGCCGTTACAACGACCTTACTAGGCTGATT  729
             ||||||||||||||||||||||||||||||||||||||||||||||||||||||||||||
Sbjct  541   AGATGGGGATTCGATGCTGCAACCATCAATAGCCGTTACAACGACCTTACTAGGCTGATT  600

Query  730   GGAAACTACACCGACTACGCTGTTCGTTGGTACAACACTGGCTTGGAGCGTGTCTGGGGT  789
             ||||||||||||||||||||||||||||||||||||||||||||||||||||||||||||
Sbjct  601   GGAAACTACACCGACTACGCTGTTCGTTGGTACAACACTGGCTTGGAGCGTGTCTGGGGT  660

Query  790   CCTGATTCTAGAGATTGGGTGAGATACAACCAGTTCAGGAGAGAATTGACCCTCACAGTT  849
             ||||||||||||||||||||||||||||||||||||||||||||||||||||||||||||
Sbjct  661   CCTGATTCTAGAGATTGGGTGAGATACAACCAGTTCAGGAGAGAATTGACCCTCACAGTT  720

Query  850   TTGGACATTGTGGCTCTCTTCAGCAACTATGACTCCAGACGTTACCCTATCCGTACAGTG  909
             ||||||||||||||||||||||||||||||||||||||||||||||||||||||||||||
Sbjct  721   TTGGACATTGTGGCTCTCTTCAGCAACTATGACTCCAGACGTTACCCTATCCGTACAGTG  780

Query  910   TCCCAACTTACCAGAGAAATCTACACTAACCCAGTTCTTGAGAACTTCGACGGTAGCTTC  969
```

```
                ||||||||||||||||||||||||||||||||||||||||||||||||||||||||||||
Sbjct  781      TCCCAACTTACCAGAGAAATCTACACTAACCCAGTTCTTGAGAACTTCGACGGTAGCTTC  840

Query  970      CGTGGTATGGCCCAGAGGATCGAACAGAACATCAGGCAGCCACACTTGATGGACATCTTG  1029
                ||||||||||||||||||||||||||||||||||||||||||||||||||||||||||||
Sbjct  841      CGTGGTATGGCCCAGAGGATCGAACAGAACATCAGGCAGCCACACTTGATGGACATCTTG  900

Query  1030     AACAGCATAACTATCTACACCGATGTGCACAGAGGATACTATTACTGGTCTGGACACC-G  1087
                ||||||||||||||||||||||||||||||||||||||||||||||||||||||||||||
Sbjct  901      AACAGCATAACTATCTACACCGATGTGCACAGAGGATACTATTACTGGTCTGGACACCAG  960

Query  1088     ATCACCGC-TCTCCAGTTGGATTC-TCCGGACCTG  1122
                |||||||||||||||||||||||| ||||||||||
Sbjct  961      ATCACCGCCTCTCCAGTTGGATTCCTCCGGACCTG  994
```

2. Comparison of reverse primers sequence and *cry1Ac-M#2* gene (target sequence used for transformation)

•

```
Score = 1779 bits (963)     Expect = 0.0    Identities = 980/987 (99%) Gaps =
6/987 (1%)            Strand=Plus/Minus

Query  145      TTAAAGATTGTACTCAGCCTCAAGAGTGGCAGTAACAGGAATGAACTCGAATCTGTCAAT  204
                ||||||||||||||||||||||||||||||||||||||||||||||||||||||||||||
Sbjct  1857     TTAAAGATTGTACTCAGCCTCAAGAGTGGCAGTAACAGGAATGAACTCGAATCTGTCAAT  1798

Query  205      GATCACTCCTGCAGTACCGCTGAAATTCCTAACACCCACGATGTTGCCCAATGAAGAAGT  264
                ||| ||||||||||||||||||||||||||||||||||||||||||||||||||||||||
Sbjct  1797     GATGACTCCTGCAGTACCGCTGAAATTCCTAACACCCACGATGTTGCCCAATGAAGAAGT  1738

Query  265      GAATGCGTTGGCACTTTCGAAGTAACCAAAATCGCTGGATTGAAGATTATCGAGTGAGGT  324
                ||||||||||||||||||||||||||||||||||||||||||||||||||||||||||||
Sbjct  1737     GAATGCGTTGGCACTTTCGAAGTAACCAAAATCGCTGGATTGAAGATTATCGAGTGAGGT  1678

Query  325      AGCAGTAGCTGGAACGGTGTTGGAGAAGATAGATGAATTGCCCCAGTTCACGTTAAGGTG  384
                ||||||||||||||||||||||||||||||||||||||||||||||||||||||||||||
Sbjct  1677     AGCAGTAGCTGGAACGGTGTTGGAGAAGATAGATGAATTGCCCCAGTTCACGTTAAGGTG  1618

Query  385      GATTGGGGTCACAGAGGCGTATCTAACCCTAACTCTGTATCTAGTAGATGTGGATGGGAA  444
                ||||||||||||||||||||||||||||||||||||||||||||||||||||||||||||
Sbjct  1617     GATTGGGGTCACAGAGGCGTATCTAACCCTAACTCTGTATCTAGTAGATGTGGATGGGAA  1558

Query  445      GTGGATAGGAACTTCGATGTAGCCTCTATTCTGAATGTTGTTGCCAGAGCTGTTAAGTCT  504
                ||||||||||||||||||||||||||||||||||||||||||||||||||||||||||||
Sbjct  1557     GTGGATAGGAACTTCGATGTAGCCTCTATTCTGAATGTTGTTGCCAGAGCTGTTAAGTCT  1498

Query  505      CACAAGATCTCCGCCAGTGAATCCTGGTCCGCTGATAACGCTTCCATTGAAAAGGAAGTT  564
                ||||||||||||||||||||||||||||||||||||||||||||||||||||||||||||
Sbjct  1497     CACAAGATCTCCGCCAGTGAATCCTGGTCCGCTGATAACGCTTCCATTGAAAAGGAAGTT  1438

Query  565      TCCCTTCACGGCAGGGATTTGAGTAATACTATCAGATGCGATGATGTTGTTGAACTCGGC  624
                ||||||||||||||||||||||||||||||||||||||||||||||||||||||||||||
Sbjct  1437     TCCCTTCACGGCAGGGATTTGAGTAATACTATCAGATGCGATGATGTTGTTGAACTCGGC  1378

Query  625      AGAACGGTGCTGCCAAGAGAAGGTAGGAGCTCTGATGATGCTCACGGAACTGTTGCTGAA  684
                ||||||||||||||||||||||||||||||||||||||||||||||||||||||||||||
Sbjct  1377     AGAACGGTGCTGCCAAGAGAAGGTAGGAGCTCTGATGATGCTCACGGAACTGTTGCTGAA  1318

Query  685      TCCGGAACGGAACATGGACACGTGGCTAAGCCTGTGGGAGAATCCAGCCCTGGGTGGCAC  744
                ||||||||||||||||||||||||||||||||||||||||||||||||||||||||||||
Sbjct  1317     TCCGGAACGGAACATGGACACGTGGCTAAGCCTGTGGGAGAATCCAGCCCTGGGTGGCAC  1258

Query  745      GCTGTTATCCTGTGGTGGGATCACGTCCAAGGAATCAACGGTTCCCCTCTGTCTGTAGAT  804
                ||||||||||||||||||||||||||||||||||||||||||||||||||||||||||||
Sbjct  1257     GCTGTTATCCTGTGGTGGGATCACGTCCAAGGAATCAACGGTTCCCCTCTGTCTGTAGAT  1198

Query  805      GGTGGATGGCAAGTTAGAAGAGGTTCCATAGGCGAACTCTGTTCCGTCAAGAACGAAAAG  864
```

```
                ||||||||||||||||||||||||||||||||||||||||||||||||||||||||||||
Sbjct  1197     GGTGGATGGCAAGTTAGAAGAGGTTCCATAGGCGAACTCTGTTCCGTCAAGAACGAAAAG  1138

Query  865      CTCCTGGTTGTTAGGACCGATATTGAAGGGTCTTCTGTACAAGGTGGAAGACAAGGTTCT  923
                ||||||||||||||||||||||||||||||||||||||||||||||||||||||||||||
Sbjct  1137     CTCCTGGTTGTTAGGACCGATATTGAAGGGTCTTCTGTACAAGGTGGAAGACAAGGTTCT  1078

Query  924      GAAGATACCGAGTCCGGTGAGGCTAACGATACGTTGTTGTGGAGCGGCGTTTCCAGCGTT  983
                ||||||||||||||||||||||||||||||||||||||||||||||||||||||||||||
Sbjct  1077     GAAGATACCGAGTCCGGTGAGGCTAACGATACGTTGTTGTGGAGCGGCGTTTCCAGCGTT  1018

Query  984      TCCGAAGAGAGGAAAAGCAAACTCAGGTCCGGAGAATCCAACTGGAGAGGCGGTGATCTG  1042
                ||||||||||||||||||||||||||||||||||||||||||||||||||||||||||||
Sbjct  1017     TCCGAAGAGAGGAAAAGCAAACTCAGGTCCGGAGAATCCAACTGGAGAGGCGGTGATCTG  958

Query  1043     GTGTCCAGACCAGTA-TAGTATC-TCTGTGCACATCGGTGTAGATAGTTATGCTGTTCA-  1099
                ||||||||||||||| ||||||| ||||||||||||||||||||||||||||||||||| 
Sbjct  957      GTGTCCAGACCAGTAATAGTATCCTCTGTGCACATC-GTGTAGAT-GTTATGCTGTTCAA  898

Query  1100     GATGTCCATCA-GTGTGGCTGCCTGAT  1125
                ||||||||||| |||||||||||||||
Sbjct  897      GATGTCCATCAAGTGTGGCTGCCTGAT  871
```

Query: DNA interspersed sequences; *Sbjct*: DNA sequences on GenBank

Table S2. Results of the comparing of interspersed sequence with GenBank sequence available

**Accession**	**Description**	**Max score**	**Total score**	**Query coverage**	**E value**	**Max ident**
AY126450.1	Synthetic construct insecticidal protein (*Cry1Ac1*) gene	3086	3086	100%	0.0	96%
Y09787.1	B. Thuringiensis *Cry1A(c)* gene	2909	2909	100%	0.0	94%
GU583853.1	Gosypium hirsutum transgenic cultivar Xinmian 33B insect-resistant	2543	2543	99%	0.0	91%
GU583854.1	Gossypium hirsutum transgenic cultivar GK-12 insect-resistant gene	2542	2542	99%	0.0	91%

Sequence of *cry1A(c)-M#2* identified similar 96% to *cry1Ac* gene (accession No. AY126450.1) on GenBank.

Table S3. Sequences of *bar* and *cry1Ac-M#2* genes using to test putative transgenic plants

Primers	Nucleotide of primers sequence (5' - 3')	Tm (^{o}C)	Size of PCR product (bp)
bar – F	TCCGTACCGAGCCGCAGGAA	55	408
bar – R	CCGGCAGGCTGAAGTCCAGC		
cry1Ac -M#2-F	ACGTTATTGTGGAGCGGCGT	55	544
cry1Ac-M#2-R	CCTCAGCGTGCTTCGAGACGT		

Table S4. Evaluation of soybean transgenic lines with *bar* and *cry1Ac*-M#2 in T0 generation after tested phosphothricin (PPT)-resistance.

No	Lines	PCR confirmation	
		bar	***cry1A(c)-M#2***
1	T_{0-1}	-	-
2	T_{0-2}	-	-
3	T_{0-3}	+	+
4	T_{0-4}	-	-
5	T_{0-5}	+	+
6	T_{0-6}	+	+
7	T_{0-7}	+	+
8	T_{0-8}	+	+
9	VX93 (Non-transgenic)	-	-

(+) positive, (-) non-positive

Supplementary Figures

Fig S1. Nucleotides sequence of *cry1Ac* mutant (M#2). Two nucleotides mutation is like TG indicated by red colorful. The note of start sequences of forward primer (arrow green color) and reverse primer (arrow red color).

Fig S2. Construction map of transfer vector, pOB-*Mut-cry1Ac*, expressing mutant *cry1Ac* with polyhedrin. The mutant fragments (821 bp) cassette digested with restriction endonucleases from the pIM-*Mut-cry1Ac* gene inserted into the pOB-*Mod-cry1Ac* to obtain the transfer vector pOB-*Mut-cry1Ac*.

Fig S3. Schematic of *cry1Ac-M#2* gene cloning using pENTR vector. The *cry1Ac-M#2* gene was cloned from the pOB-Mut-cry1Ac vector by PCR with the specific primers cry1Ac –F/R. The PCR products were attached directly into the cloning vector pENTR TM/D – TOPO by ligase reaction.

Fig S4. Bacteria *A. tumefaciens* strain carrying vector pB2GW7-cry1Ac-M#2. The *cry1Ac-M#2* gene was transferred from the cloning vector pENTRTM/D-TOPO-cry1A(c) to the pB2GW7 vector by LR reaction. Plasmid harboring pB2GW7- cry1Ac-M#2 was transferred into *A. tumefaciens EHA105* by electrical impulse method.

Fig S5. Isolation of *cry1A(c)-M#2* product. (A) Cloning *cry1A*(c)-M#2 derived from pOB-*cry1A*(c)-M#2 vector by PCR, with control (lane 1) and pOB-*cry1A*(c)-M#2 vector (lane 2). (B) Results in confirmation of *cry1A*(c)-M#2 gene in randomly *E.coli* colonies harbored *pB2GW7-cry1A*(c)-M#2 recombinant vector (lane 2 to 13), lane 1 is control (water). (C) Result in the confirmation of *cry1A*(c)-M#2 gene in *pB2GW7* vector cut by *SacI* enzyme from twelfth colonies, control is *pB2GW7* vector non-cut. Marker 1 kb used in the experiment from Bioneer.
