# Supplementary material for: Convergence of Bar and Cry1Ac Mutant Genes in Soybean Confers Synergistic Resistance to Herbicide and Lepidopteran Insects
Source: Front Plant Sci. 2021 Oct 14;12:698882. doi: 10.3389/fpls.2021.698882 (PMC8559871; doi:10.3389/fpls.2021.698882)
Supplement: Supplementary file 2 [file Data_Sheet_2.pdf]

Mutant #2 (Mut-2)

ATGGACAACAACCCAAACATCAACGAATGCATTCCATACAACTGCTTGAGTAACCCAGAAGTTGA
AGTACTTGGTGGAGAACGCATTGAAACCGGTTACACTCCCATCGACATCTCCTTGTCCTTGACAC
AGTTTCTGCTCAGCGAGTTCGTGCCAGGAGCTGGGTTCGTTCTCGGACTAGTTGACATCATCTGG
GGTATCTTTGGTCCATCTCAATGGGATGCATTCCTGGTGCAAATTGAGCAGTTGATCAACCAGAG
GATCGAAGAGTTCGCCAGGAACCAGGCCATCTCTCGTTTGGAAGGATTGAGCAATCTCTACCAAA
TCTATGCAGAGAGCTTCAGAGAGTGGGAAGCCGATCCTACTAACCCAGCTCTCCGCGAGGAAATG
CGTATTCAATTCAACGACATGAACAGCGCCTTGACCACAGCTATCCCATTGTTCGCAGTCCAGAA
CTACCAAGTTCCTCTCTTGTCCGTGTACGTTCAAGCAGCTAATCTTCACCTCAGCGTGCTTCGAG
ACGTTAGCGTGTTTGGGCAAAGATGGGGATTCGATGCTGCAACCATCAATAGCCGTTACAACGAC
CTTACTAGGCTGATTGGAAACTACACCGACTACGCTGTTCGTTGGTACAACACTGGCTTGGAGCG
TGTCTGGGGTCCTGATTCTAGAGATTGGGTGAGATACAACCAGTTCAGGAGAGAATTGACCCTCA
CAGTTTTGGACATTGTGGCTCTCTTCCCGAACTATGACTCCAGACGTTACCCTATCCGTACAGTG
TCCCAACTTACCAGAGAAATCTACACTAACCCAGTTCTTGAGAACTTCGACGGTAGCTTCCGTGG
TTCTGCCCAGGGTATCGAAAGATCCATCAGGAGCCCACACTTGATGGACATCTTGAACAGCATAA
CTATCTACACCGATGTGCACAGAGGATACTATTACTGGTCTGGACACCAGATCATGGCCTTTCCA
GTTGGATTCTCCGGACCTGAGTTTACCTTTCCTCTCTATGGAACTATGGGAAACGCCGCTCCACA
ACAACGTATCGTTGCTCAACTAGGACAGGGTGTCTACAGAACCTTGTCTTCCACCTTGTACAGAA
GACCCTTCAATATCGGTATCAACAACCAGCAACTTTCCGTTCTTGACGGAACAGAGTTCGCCTAT
GGAACCTCTTCTAACTTGCCATCCGCTGTTTACAGAAAGAGCGGAACCGTTGATTCCTTGGACGT
GATCCCACCACAGAACAACAATGTGCCACCCAGGCAAGGATTCTCCCACAGGCTTAGCCACGTGT
CCATGTTCCGTTCCGGATTCAGCAACAGTTCCGTGAGCATCATCAGAGCTCCTATGTTCTCTTGG
ATTCACCGTTCTGCCGAGTTCAACAACATCATCGCATCTGATAGTATTACTCAAATCCCTGCCGT
GAAGGGAAACTTCCTTTTCAATGGAAGCGTTATCAGCGGACCAGGATTCACTGGCGGAGATCTTG
TGAGACTTAACAGCTCTGGCAACAACATTCAGAATAGAGGCTACATCGAAGTTCCTATCCACTTC
CCATCCACATCTACTAGATACAGAGTTAGGGTTAGATACGCCTCTGTGACCCCAATCCACCTTAA
CGTGAACTGGGGCAATTCATCTATCTTCTCCAACACCGTTCCAGCTACTGCTACCTCACTCGATA
ATCTTCAATCCAGCGATTTTGGTTACTTCGAAAGTGCCAACGCATTCACTTCTTCATTGGGCAAC
ATCGTGGGTGTTAGGAATTTCAGCGGTACTGCAGGAGTGATCATTGACAGATTCGAGTTCATTCC
TGTTACTGCCACTCTTGAGGCTGAGTACAATCTTTAA

ACCGATGTGCACAGAG

Fig S1. Nucleotides sequence of *cry1Ac* mutant (M#2). Two nucleotides mutation is like TG indicated by red colorful. The note of start sequences of forward primer (arrow green color) and reverse primer (arrow red color).

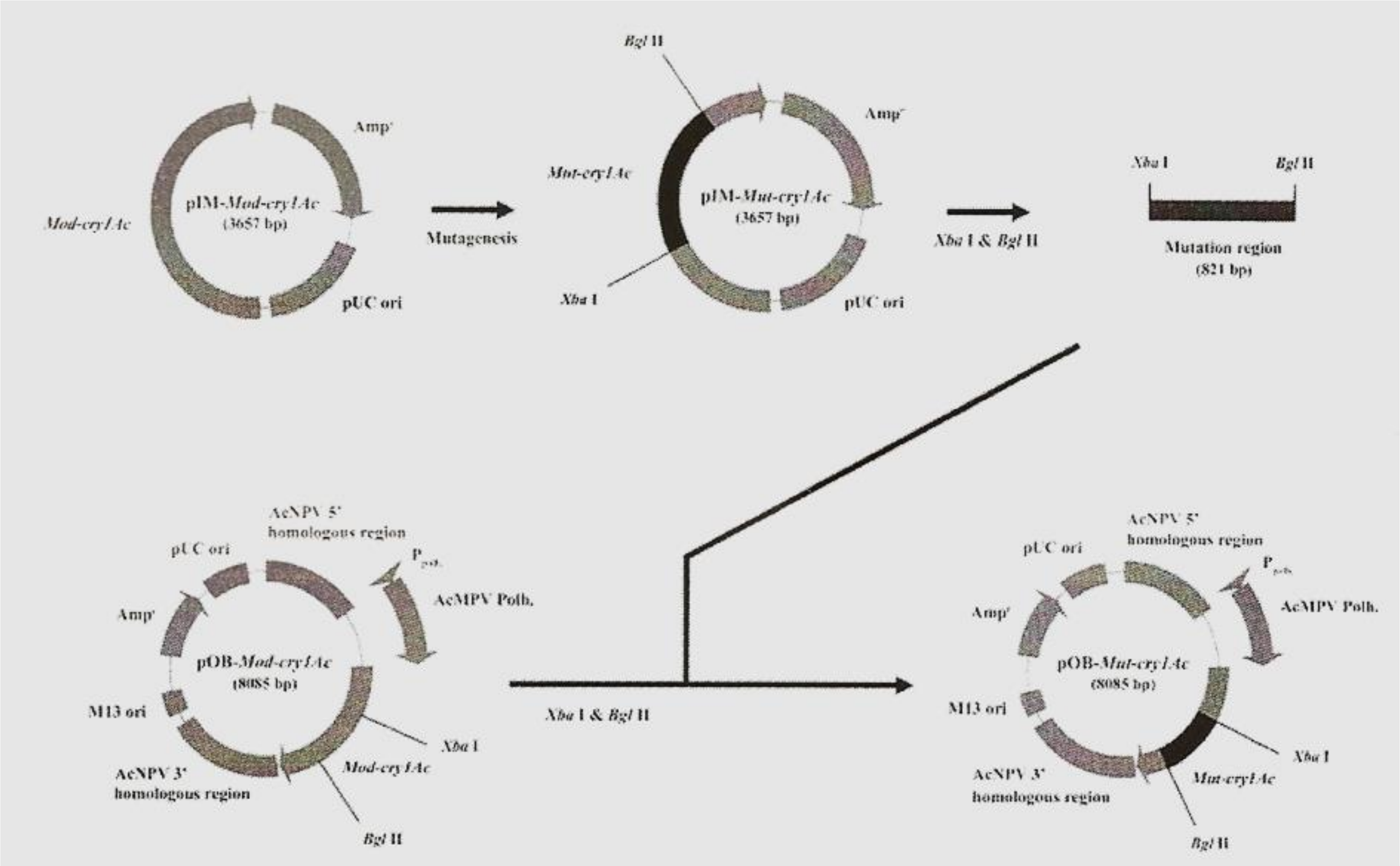

Fig S2. Construction map of transfer vector, pOB-*Mut-cry1Ac*, expressing mutant *cry1Ac* with polyhedrin. The mutant fragments (821 bp) cassette digested with restriction endonucleases from the pIM-*Mut-cry1Ac* gene inserted into the pOB-*Mod-cry1Ac* to obtain the transfer vector pOB-*Mut-cry1Ac*.

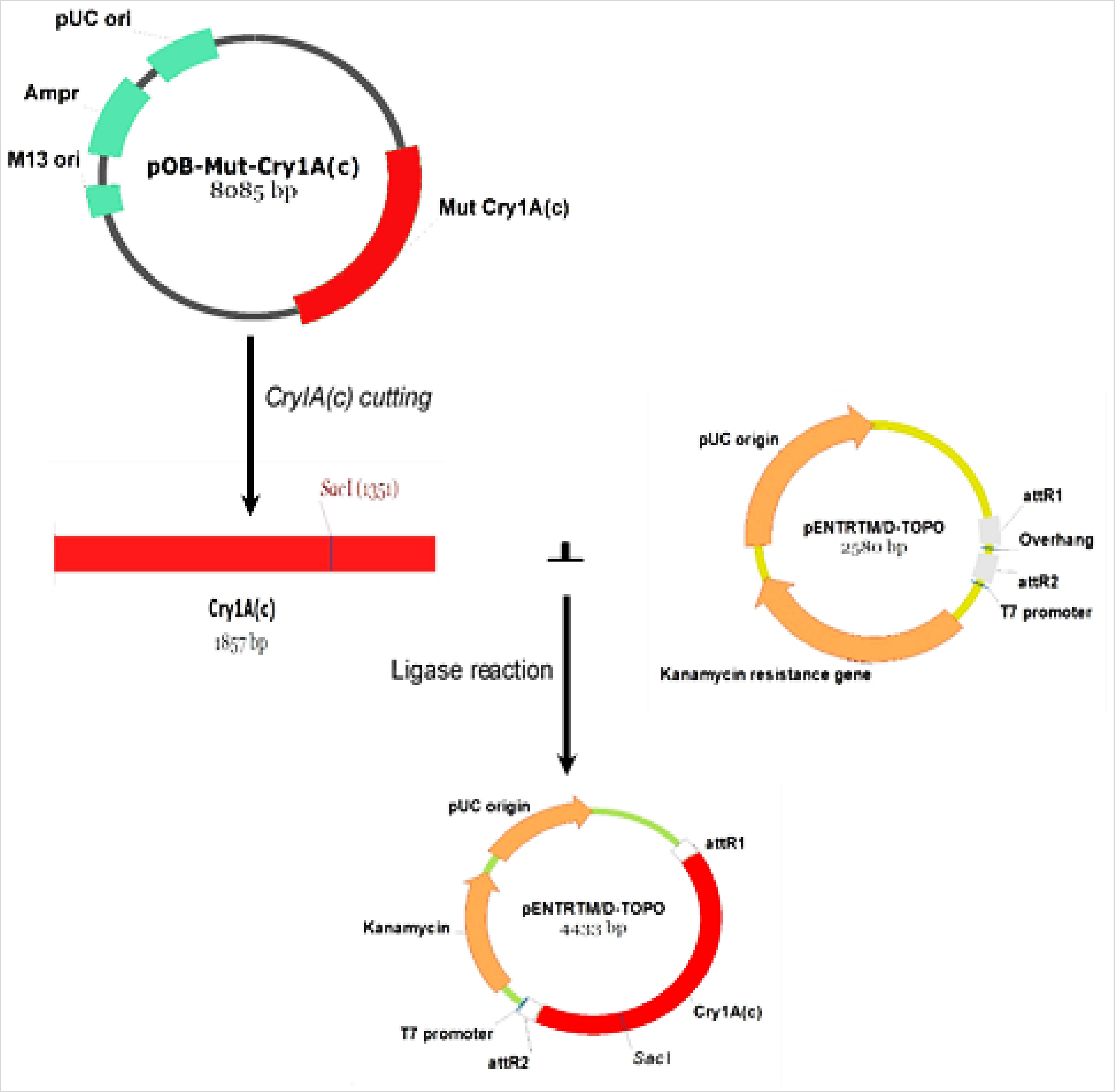

Fig S3. Schematic of *cry1Ac-M#2* gene cloning using pENTR vector. The *cry1Ac-M#2* gene was cloned from the pOB-Mut-cry1Ac vector by PCR with the specific primers cry1Ac –F/R. The PCR products were attached directly into the cloning vector pENTR TM/D – TOPO by ligase reaction.

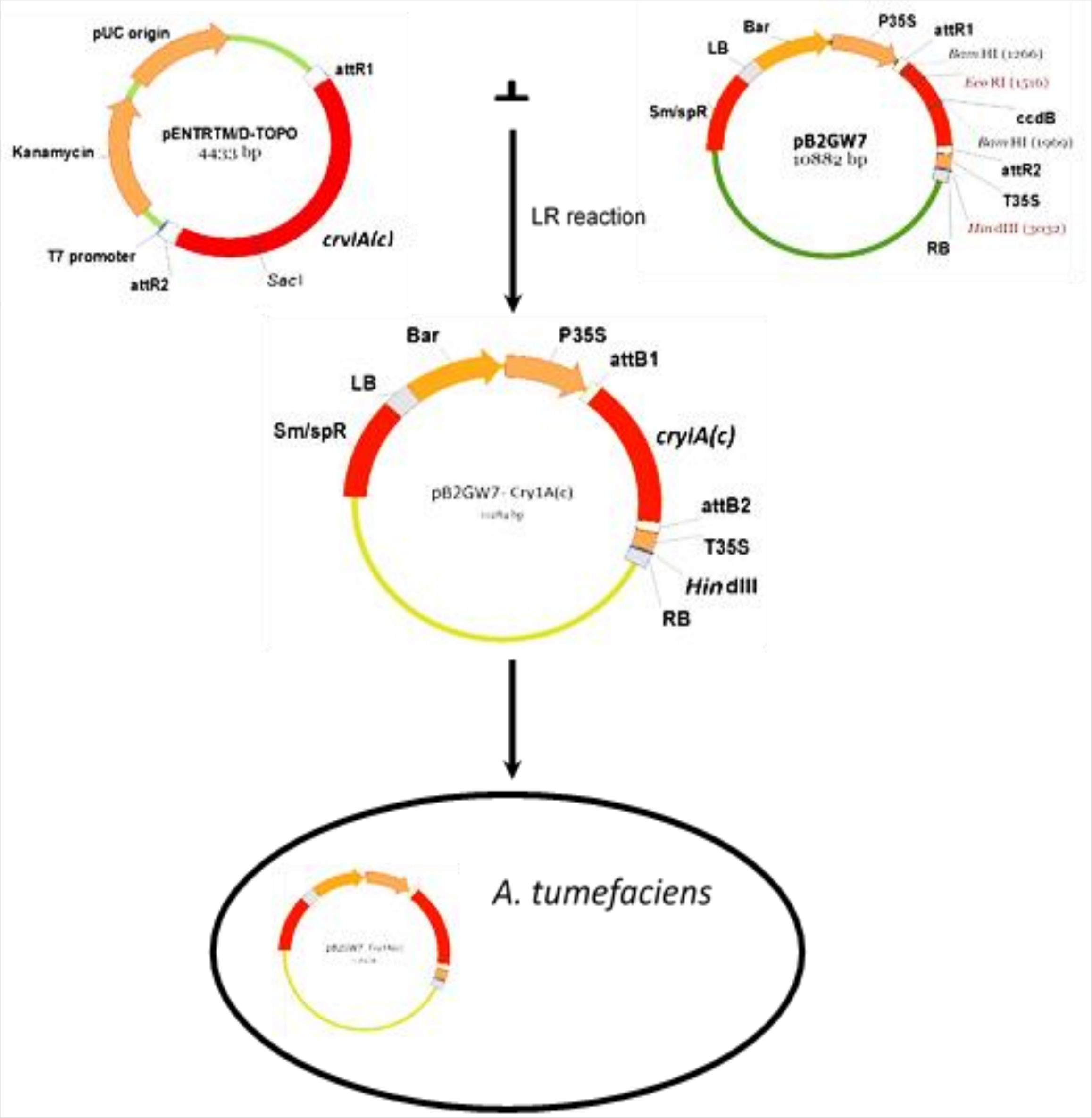

Fig S4. Bacteria *A. tumefaciens* strain carrying vector pB2GW7-cry1Ac-M#2. The *cry1Ac-M#2* gene was transferred from the cloning vector pENTRTM/D-TOPO-cry1A(c) to the pB2GW7 vector by LR reaction. Plasmid harboring pB2GW7- cry1Ac-M#2 was transferred into *A. tumefaciens EHA105* by electrical impulse method.

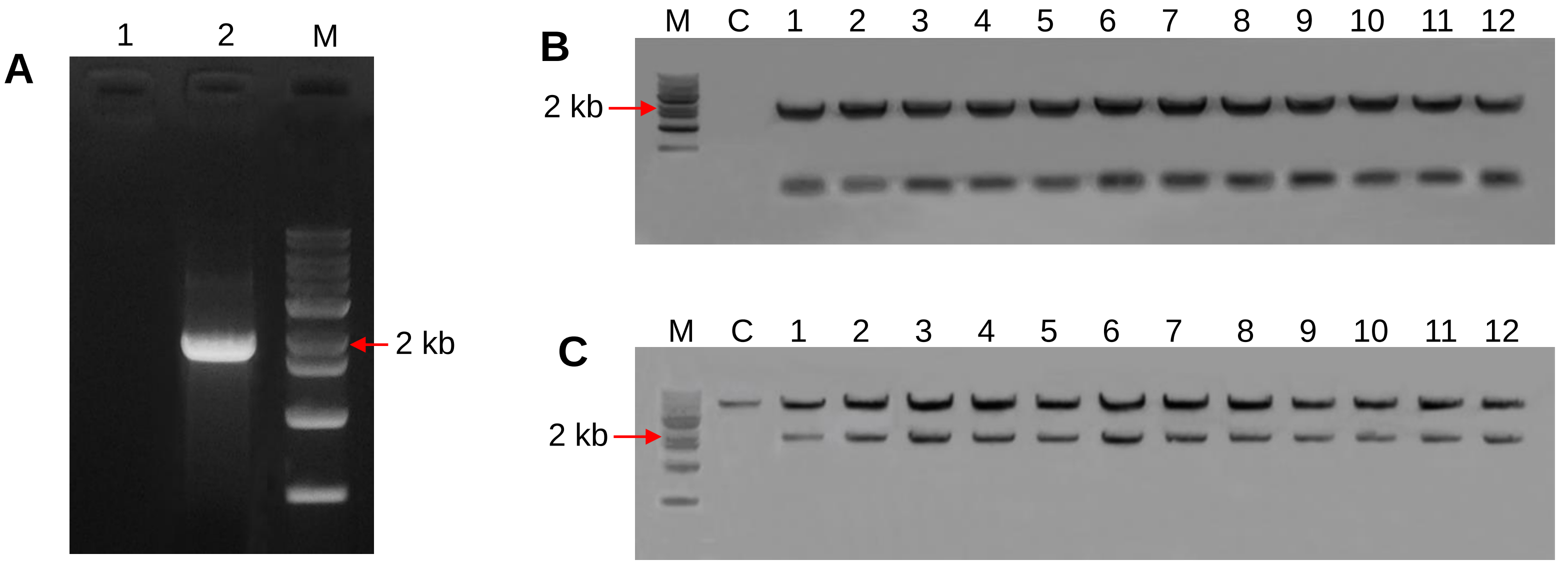

Fig. S5. Isolation of *cry1A(c)-M#2* product. (A) Cloning *cry1A*(c)-M#2 derived from pOB-*cry1A*(c)-M#2 vector by PCR, with control (lane 1) and pOB-*cry1A*(c)-M#2 vector (lane 2). (B) Results in confirmation of *cry1A*(c)-M#2 gene in randomly E.coli colonies harbored *pB2GW7-cry1A*(c)-M#2 recombinant vector (lane 2 to 13), lane 1 is control (water). (C) Result in the confirmation of *cry1A*(c)-M#2 gene in *pB2GW7* vector cut by *SacI* enzyme from twelfth colonies, control is *pB2GW7* vector non-cut. Marker 1kb used in the experiment from Bioneer.
